# A review of methods for addressing components of interventions in meta-analysis

**DOI:** 10.1371/journal.pone.0246631

**Published:** 2021-02-08

**Authors:** Maria Petropoulou, Orestis Efthimiou, Gerta Rücker, Guido Schwarzer, Toshi A. Furukawa, Alessandro Pompoli, Huiberdina L. Koek, Cinzia Del Giovane, Nicolas Rodondi, Dimitris Mavridis

**Affiliations:** 1 Faculty of Medicine and Medical Center, Institute of Medical Biometry and Statistics, University of Freiburg, Freiburg, Germany; 2 Department of Primary Education, Evidence Synthesis Methods Team, University of Ioannina, Ioannina, Greece; 3 Institute of Social and Preventive Medicine (ISPM), University of Bern, Bern, Switzerland; 4 Department of Psychiatry, University of Oxford, Oxford, United Kingdom; 5 Departments of Health Promotion and Human Behavior and Clinical Epidemiology, Kyoto University Graduate School of Medicine/School of Public Health, Kyoto, Japan; 6 Psychiatric Rehabilitation Clinic Villa San Pietro, Trento, Italy; 7 Department of Geriatric Medicine, University Medical Centre Utrecht, Utrecht University, Utrecht, The Netherlands; 8 Institute of Primary Health Care (BIHAM), University of Bern, Bern, Switzerland; 9 Population Health Laboratory (#PopHealthLab), University of Fribourg, Fribourg, Switzerland; 10 Department of General Internal Medicine, Inselspital, Bern University Hospital, University of Bern, Bern, Switzerland; 11 Faculté de Médecine, Paris Descartes University, Sorbonne Paris Cité, Paris, France; Witten/Herdecke University, GERMANY

## Abstract

Many healthcare interventions are complex, consisting of multiple, possibly interacting, components. Several methodological articles addressing complex interventions in the meta-analytical context have been published. We hereby provide an overview of methods used to evaluate the effects of complex interventions with meta-analytical models. We summarized the methodology, highlighted new developments, and described the benefits, drawbacks, and potential challenges of each identified method. We expect meta-analytical methods focusing on components of several multicomponent interventions to become increasingly popular due to recently developed, easy-to-use, software tools that can be used to conduct the relevant analyses. The different meta-analytical methods are illustrated through two examples comparing psychotherapies for panic disorder.

## Introduction

Complex interventions are increasingly employed in public health. Several definitions are provided in the literature for complex interventions [1–4]. Such interventions are usually multifaceted, i.e. comprise several, potentially active and possibly interacting components (multicomponent interventions).

Several articles are discussing methodological challenges at each stage of a systematic review with complex interventions published in the *Journal of Clinical Epidemiology* in 2013 and the *Agency for Healthcare Research and Quality (AHRQ) Series on Complex Intervention Systematic Reviews* [1, 5–18]. Furthermore, a special series of seven articles were recently published in the *British Medical Journal Global Health* considering challenges in evidence synthesis with complex interventions under the World Health Organization (WHO) guideline development [19–25].

Evaluating the effects of multi-component interventions requires tailored statistical synthesis methods. For example, consider a randomized controlled trial (RCT) on interventions for weight loss, in which a group of people is randomized to follow a certain diet and physical exercise while another group is randomized to a placebo diet. The intervention group consists of two components, diet, and physical exercise, and can be regarded as a complex intervention. We could easily estimate the relative effect of this complex intervention versus placebo. However, it might be of interest to disentangle the individual effects of the intervention components (diet and physical exercise). This question cannot be answered by this particular RCT design, but it could be estimated using a factorial RCT where participants are allocated to receive neither intervention, one or the other, or both [26]. Alternatively, we can estimate the component effect using appropriate meta-analytical methods if there exist other studies that compare the diet versus placebo, and studies that compare physical exercise versus placebo.

More generally, components may act independently, synergistically (i.e. the effect of their combination is larger than the sum of their individual effects), or even antagonistically. In this article, we provide a review of the methodology regarding meta-analytical approaches for evaluating the effects of complex interventions. We focus more on component network meta-analysis (CNMA), which allows estimating the component effects of several multicomponent interventions [27]. We exemplify the identified meta-analytical methodologies through their implementation in systematic reviews of psychological interventions for panic disorder and discuss their advantages and disadvantages.

## Materials and methods

We searched in the literature for methodological articles that address the effects of complex interventions with meta-analytical models. We also searched in a database for published papers regarding network meta-analysis with respect to multicomponent interventions [28]. By inspecting those identified published papers that employ complex interventions and references therein, and based on our expertise on the subject, we provide an overview of meta-analytical methods that address the effects of complex interventions.

### Meta-analytical models evaluating the effects of complex interventions

We identified twelve articles that present meta-analysis models for evaluating the effects of multicomponent interventions or providing methodological aspects for their implementation (Table 1). These twelve articles could be categorized in one or more than one of the below categories according to their methodological content: (1) provide methodological challenges of an existing meta-analytic method to deal with complex interventions (1 article); (2) present or extend a novel meta-analytical model (or framework) to deal with complex interventions (5 articles); (3) provide methods/ simulation/ prerequisites to assess the assumption of models (3 articles); (4) methodological review of meta-analytical models addressing complexity (3 articles); and model selection for component network meta-analysis (1 article) (Table 1). Table 2 presents an overview of the methods, outlining the possible benefits and limitations of each meta-analytical model identified.

**Table 1:** Categorization of the twelve methodological articles with meta-analysis models that evaluate the effects of complex interventions.

Categorization of articles according to methodology content	Number of Articles*	Details of Articles (authors, year of publication, reference) *
Provide methodological challenges of existing meta-analytic methods to deal with complex interventions	1	Jonkman *et al*. (2017) [29]
Present or extend a novel meta-analytical approach (or framework) to deal with complex interventions	5	Welton *et al*. (2009) [27] Madan *et al*. (2014) [30] Bangdiwala *et al*. (2018) [31] Freeman *et al*. (2018) [32] Rücker, Petropoulou, and Schwarzer (2020)* [33]
Provide methods/ simulation/ prerequisites to assess the assumption of models	3	Thorlund and Mills (2012) [34] Mills, Thorlund, and Ioannidis (2012) [35] Rücker, Petropoulou, and Schwarzer (2020)* [33]
A methodological review of meta-analytical models addressing the complexity	3	Caldwell and Welton (2016) [36] Tanner-Smith and Grant (2018) [37] Higgins *et al*. (2019) [23]
Model selection for component network meta-analysis	1	Rücker, Schmitz, and Schwarzer (2020) [38]

*One article was classified into two categories.

**Table 2:** Description, benefits, and limitations of the meta-analytical approaches that evaluate the effects of complex interventions.

	Meta-analytic approach	Description	Benefits	Limitations
	**Pairwise Standard Meta-analysis**	Each observed different combination of components is classified as either active or control. A pairwise meta-analysis is conducted to assess the effectiveness of the two groups.	Easy to estimate the intervention effect. Answers the question ‘Are active interventions (which may include different combinations of components) effective on average?’ Increased statistical power and reduced possibility of false positives.	Meta-analysis with heterogeneous studies may be less interpretable and generalizable than initially anticipated. Cannot identify which components or combinations thereof are effective.
	**Subgroup analysis**	Interventions are grouped firstly into clinically meaningful, prespecified groups, and afterward subgroup analyses are conducted.	Possible to investigate the impact of major contextual influences (such as different implementation mechanisms).	Subgroup analysis typically provides low power to detect reliable results. Risk of increasing the type I error rate through multiple testing. Difficulties to interpret the results of several pairwise meta-analyses. Difficult to implement subgroup analysis when studies are comparing active interventions (not a star network).
	**Meta-regression (for two interventions)**	Components of complex interventions enter the model as regressors.	Can answer the question ‘Which component regressor is most effective?’ Investigates sources of heterogeneity; e.g. explores how effectiveness changes with the inclusion or exclusion of certain components.	Requires a reasonably large number of studies for reliable results; has low power. Difficult to implement meta-regression when studies are comparing active interventions (not a star network).
	**Component individual participant data (IPD) meta-analysis**	IPD are retrieved and the two- or the one-stage approach of individual participant data meta-analysis is conducted.	Component IPD meta-analysis allows investigating component effect heterogeneity. Component IPD allows investigating the interaction between components and participant-level characteristics. Causal mechanisms in component IPD meta-analysis can help to further determine which characteristics of complex interventions work best in which patient subgroups.	IPD is rarely available. May be subject to data availability bias. Delays and difficulties in getting the IPD.
**Meta-regression for more than two interventions**	**Standard network meta-analysis (NMA) Full interaction ΝΜΑ**	Each combination of components seen in the data is considered to be a separate intervention and is assigned its own effect.	Estimates the effectiveness of each combination of components observed in the data and provides a hierarchy of the combination of components.	The model can only address the effectiveness of the observed combination of components. Low statistical power. More parameters are estimated compared to additive and interaction component NMA models. Each study may include each own set of interventions (i.e. unique combination of components). Subsets of components may appear in both the most and least effective interventions, making interpretation challenging. The model is unusable when the data comprises multiple disconnected networks.
	**Additive CNMA model Component NMA (CNMA)**	Assumes that each component has a separate independent effect. The total effect of an intervention is equal to the sum of the component effects (additivity assumption).	Estimates the effectiveness of each component and provides a hierarchy of components. Addresses the questions “Which of all possible combinations of components is more effective?” and “What components should an intervention include?”.	If the additivity assumption is mildly or strongly violated, the estimates of component effects may be biased. Interactions between components are ignored. Errors in the definition of components may compromise results.
	**Interaction CNMA model Component NMA (CNMA)**	Allows two or more pairs (or trios/ quadruples etc.) of components to have a larger or smaller effect (synergistic/ antagonistic) than the sum of their effects. It extends the additive CNMA model.	Can answer the question “Which of all possible combinations of components is most effective, while accounting for a specified interaction between the components?”. The same benefits with the additive CNMA model but additionally allows for interactions between components. Interaction terms should be defined a-priori and model selection methods can be used on top of this prespecification to decide which interactions to include in the model.	The same limitations with the additive CNMA model but interactions between components are not ignored. More parameters are estimated compared to the additive CNMA model. Difficult to prespecify interaction terms using clinical or statistical perceptive.

### Standard meta-analytical approaches comparing two interventions (meta-analysis, subgroup analysis, meta-regression)

Caldwell and Welton described standard meta-analysis in the context of complex intervention as the ‘lumping approach’ [36]. In a standard pairwise meta-analysis, all active interventions are grouped as a single ‘intervention’ against different kinds of ‘no intervention’ (e.g. placebo, usual intervention, waiting list, no intervention) that grouped as a control (reference intervention) (Table 2). This is the simplest model answering the clinical question ‘Are active interventions (on average) effective?’. This is very important if we are interested in obtaining an estimate of the overall effectiveness of active interventions.

**Fig 1:**
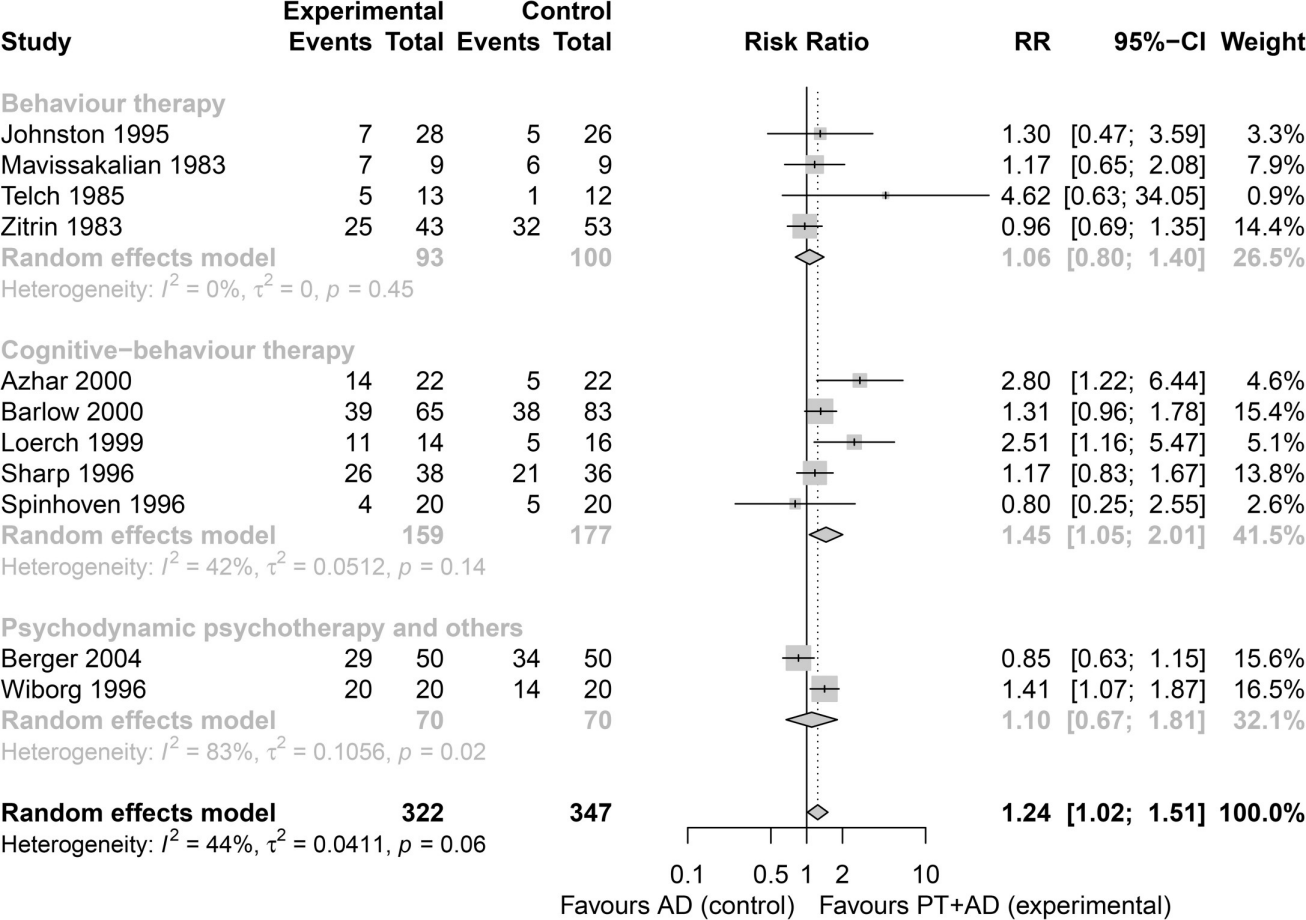
Forest plot for a subgroup of studies that compared combined PT+AD versus AD for the rate of response (i.e. substantial improvement) at 2–4 months in patients with panic disorder with or without agoraphobia. Subgroup analysis is conducted for the three components of the combined psychotherapy: (1) *Behaviour therapy*, (2) *Cognitive-behaviour therapy*, (3) *Psychodynamic psychotherapy*, *and others*. The analysis provided with the inverse-variance random-effects model with R package **meta** [41]. The risk ratio was used as an effect size. Heterogeneity was estimated with the DerSimonian-Laird estimation method.

This can be illustrated by the study of Furukawa *et al*., who conducted a synthesis of studies that compared combined psychotherapy plus antidepressants (PT+AD) versus antidepressants (AD) for the rate of response (i.e. substantial improvement) at 2–4 months in patients with panic disorder with or without agoraphobia [39]. They considered the following three clinically meaningful groups; (1) *Behaviour therapy* (BT), (2) *Cognitive-behaviour therapy* (CBT), and (3) *Psychodynamic psychotherapy and others* (PP). Subgroup analysis was employed to explore component effects (BT, CBT, PP). Fig 1 provides the forest plot for subgroup analysis of studies that compared PT+AD (experimental group) versus AD (control group) for panic disorder. The combination of PT+AD versus AD from the *Cognitive-behaviour therapy* group had the largest effect size (risk ratio = 1.45, 95% confidence interval CI [1.05, 2.01]), but pooled effects across subgroups overlapped and there was no strong evidence of a difference (test for subgroup differences: p = 0.32). A limitation of subgroup analysis is that it typically has low power and a risk of increasing the type I error rate through multiple testing [6, 40] (Table 2).

Meta-regression addresses the research question ‘Which components are effective?’ and requires a reasonably large number of studies for powerful results (Table 2). One application of meta-regression with an interest in components of complex interventions was employed by Bower *et al*., who aimed to identify ‘active ingredients’ of collaborative care interventions for depression in primary care [42]. Table 3 shows the meta-regression results for the synthesis of studies for panic disorder to compare the aforementioned three component effects (BT, CBT, PP*)* (only one is considered at a time). Here, CBT had also the largest effect size, however, there is again no strong evidence for a difference (p = 0.49).

**Table 3:** Meta-regression results for the synthesis of studies that compared combined psychotherapy plus antidepressants (PT+AD) versus antidepressants (AD) for the rate of response (i.e. substantial improvement) at 2–4 months in patients with panic disorder with or without agoraphobia [39].

Inclusion of component in the combination of PT+AD intervention	Component estimate Risk Ratio (95% CI)
Behaviour therapy	1.14 (0.75, 1.72)
Cognitive-behaviour therapy	1.46 (1.05, 2.04)
Psychodynamic psychotherapy and others	1.10 (0.75, 1.64)
Heterogeneity: *τ*^2^ = 0.058, *Q* = 15.29, *df* = 8, *p* = 0.054, *I*^2^ = 48%	

*Behaviour therapy*, *Cognitive-behaviour therapy*, and *Psychodynamic psychotherapy*, *and others* are entered as regressors in meta-regression analysis. The R package **meta** [41] was used with the risk ratio as the effect measure. Heterogeneity was estimated with the DerSimonian-Laird estimation method.

Additionally, Bangdiwala *et al*. proposed a new statistical framework for meta-regression to evaluate the effectiveness of non-randomized, dynamic complex interventions from community-based studies by modeling the observed outcome rather than the observed intervention effect [31].

### Component individual participant data meta-analysis

Individual participant data (IPD) is the gold standard in evidence synthesis [43–45]. A detailed methodology for the IPD meta-analysis model can be found in Debray *et al*. [46]. There are several advantages of having IPD compared to aggregated data, such as the explanation of potential sources of heterogeneity, updated data sets, investigation of the interaction between interventions and participant-level characteristics, and better data understanding. On the other hand, IPD are rarely available or difficult, and time-consuming to obtain [47].

General benefits and limitations of IPD meta-analysis also apply when evaluating complex interventions (Table 2). Jonkman *et al*. (Table 1) discussed methodological challenges that can be addressed when evaluating complex interventions in the IPD meta-analysis model [29]. One of the challenges is that complex interventions are typically heterogeneous and therefore a meta-regression model using IPD can be helpful to investigate how the component effects differ according to study-level characteristics (component effect heterogeneity) [29]. Jonkman *et al*. suggested the use of causal mechanisms in component IPD meta-analysis as they can help to further determine which characteristics of complex interventions work best in which patient subgroups [29].

Methodological challenges encountered in two application papers of component IPD meta-analyses provided by Jonkman *et al*. [48–51] may help researchers to carefully prepare the resource-intensive IPD meta-analyses.

### Standard Network Meta-Analysis (NMA)

Standard NMA is a weighted regression that synthesizes direct (from head-to-head experiments) and indirect evidence (obtained via a common comparator) to allow multiple intervention comparisons. Interventions are rarely identical across studies but using too narrow criteria for defining interventions may result in each study comparing a different set of interventions. In such a case, the standard NMA model may become unidentifiable, i.e. when the data forms two or more disconnected networks.

In the case of interventions consisting of multiple components, standard NMA is also referred to as *a full interaction model* and considers each combination of components seen in the data as a separate intervention. Standard NMA answers the question “Which combination of components (seen in the data) is most effective?”. Tricco *et al*. [52] provided an application article of the standard NMA model for evaluating and comparing the effectiveness of combinations of intervention components for the prevention of falls.

The number of published network meta-analyses evaluating multicomponent interventions is increasing [28, 53]. According to a database of 456 published networks of interventions provided by Petropoulou *et al*. [28], 59 (13%) networks considered multicomponent interventions; while only 5 out of these 59 networks (8%) were published in 2011, the number of networks increased to 26 (44%) in 2014.

**Fig 2:**
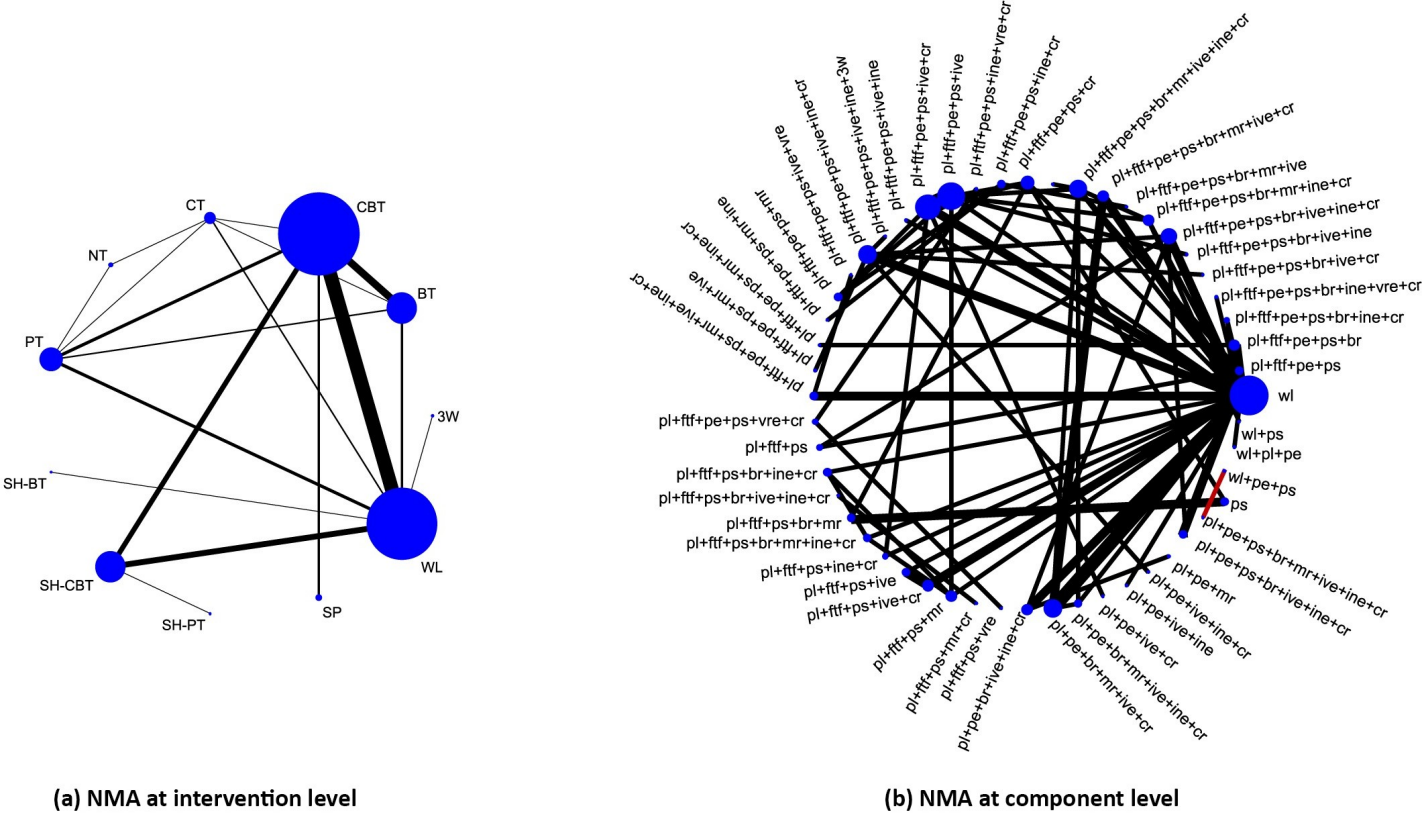
Network plot for psychological interventions at intervention and component level. On the left, we show the network plot at the intervention level (Fig 2(A)). Each circle (node) represents an intervention. Solid lines indicate comparisons for which direct information was available. Abbreviations for 11 interventions: Waiting List (WL); Supportive Psychotherapy (SP); Self Help Physiological Therapy (SH-PT); Self Help Cognitive Behavioral Therapy (SH-CBT); Self Help Behavioral Therapy (SH-BT); Physiological Therapy (PT); No Intervention (NT); Cognitive Therapy (CT); Behavioral Therapy (BT); Cognitive Behavioral Therapy (CBT); Third Wave CBT (3W)). On the right, we show the network plot at the component level (Fig 2(B)). Each node corresponds to a particular combination of components. Abbreviations for 12 components: waiting component (*wl*); placebo effect (*pl*); psychological support (*ps*); psychoeducation (*pe*); breathing retraining (*br*); progressive/applied muscle relaxation (*mr*); cognitive restructuring (*cr*); interoceptive exposure (*ine*); in vivo exposure (*ive*); virtual reality exposure (*vre*); 3w, third-wave components (*3w*); face-to-face setting (*ftf*).

A case study provided by Pompoli *et al*. [54] explored 12 components of eleven psychological interventions for panic disorder; the data are available in S1 Table. Fig 2(A) (left-hand side) provides the graphical representation of the network structure at the *intervention level* with each node denoting one of the eleven psychological interventions. At the component level, the available studies compared a total of 51 interventions; 49 combinations of components and 2 single components. If we treat each combination as a distinct intervention, we will have 50 different parameters (relative intervention effects versus a reference) to estimate, resulting in low power. Fig 2(B) shows the network plot at the *component level* with each node denoting the various combinations of components that appear in the network. Intervention comparisons between eleven psychological interventions give a connected NMA structure (Fig 2(A)), but splitting interventions into their components leads to disconnected component comparisons such as *pl+ pe+ps+br+mr+ive+ine+cr* versus *wl+pe+ps* (Fig 2(B), red edge).

Even if we were able to fit a standard NMA model (i.e. if the network was connected), combinations of component effects may be difficult to interpret in practice. For example, we may find that the relative effect of the *third-wave component (3w)*, when used as a standalone intervention, versus waiting list, is estimated with precision; but, we may also find that when the *3w component* is used in combination with other components, the estimated relative effect versus waiting list is very imprecise. This could happen when few studies combine the *3w component* with other components and we do not have enough evidence to detect an effect.

### NMA models considering components of interventions / CNMA

When all studies compare an intervention to common control, we may consider a meta-regression or subgroup analyses to explore if the effect is moderated by the type of intervention. Considering the components of interventions, CNMA allows the estimation of various component effects and all possible combinations of components [27]. Splitting interventions into different components may lead to a sparse or disconnected network. However, disconnected networks may share common components that can be used to *reconnect* the network at the component level.

In a seminal article, Welton *et al*. [27] suggested CNMA models evaluate the effects of complex interventions. The models they proposed are the additive effects CNMA model and the CNMA model with interactions. The description, benefits, and limitations of the suggested models used to handle complex interventions are shown in Table 2. A recently published article by Freeman *et al*. [32] reviewed all models presented in Welton *et al*. [27] and extended the CNMA models to account for covariates. A recently published article by Rücker *et al*. [33] described the additive and interaction CNMA models in a frequentist framework [55]. Madan *et al*. [30] employed interaction and additive CNMA model in time-to-event data by categorizing smoking cessation electronic or non-electronic interventions into five electronic and five non-electronic components.

### Additive effects CNMA model

The additive effects CNMA model assumes that each intervention effect equals the sum of the effects of the corresponding components it comprises [23, 27, 32, 33, 36]. This is known as the additivity assumption and it suggests that the relative effect of intervention *A*+*B* comprising two components *A* and *B* versus intervention C is *d*_*A*+*B vs C*_ = *d*_*A*_+*d*_*B*_−*d*_*C*_, where: *d*_*t*1 *vs t*2_ is the relative effect of intervention *t*1 versus intervention *t*2; *d*_*t*1_ the effect of intervention *t*1; and *d*_*t*2_ the effect of intervention *t*2. This model answers not only the question ‘Which of all possible combinations of components is the most effective?’ but also ‘Which components are the most effective?’ [27]. Additive CNMA models allow estimating the relative effects between components and a combination of components and can provide a hierarchy of components.

Under the additivity assumption, the effect of adding component *c* to intervention is independent of the intervention. In other words, under additivity, the relative effect of the intervention (*c*+*X*) versus intervention *X* is the same for all *X* (i.e. for *X* being any combination of components other than *c*). In essence, this model assumes that components do not interact with each other. Consider the active drugs *A*, *B*, *C*, *D*, and *E*. A study that compares (*A*+*B*+*C*) vs (*A*+*B*+*D*) estimates *C* vs *D* under the additivity assumption (and A, B components cancel out), the same with a study that compares (*E*+*C*) vs (*E*+*D*) [54]. In the panic disorder example [54], the relative effect for additive CNMA model of intervention *pl+ftf+pe+ps+ive+ine* versus intervention *wl* is *d*_*pl*+*ftf*+*pe*+*ps*+*ive*+*ine vs wl*_ = *d*_*pl*_+*d*_*ftf*_+*d*_*pe*_+*d*_*ps*_+*d*_*ive*_+*d*_*ine*_−*d*_*wl*_.

Rücker *et al*. proposed a likelihood ratio test of the additivity assumption to compare NMA and additive CNMA model [33]. Mills *et al*. [35] provided methods and prerequisites to assess the additivity assumption. Thorlund and Mills conducted a simulation study and found that if additivity holds, the additive CNMA estimates are more precise than conventional NMA estimates and the additive CNMA model is comparably advantageous than standard NMA in terms of bias when additivity is mildly violated [34]. This suggests that if additivity holds approximately, using the additive CNMA model can be beneficial [34].

### Interaction CNMA model

The interaction CNMA model is an extension of the additive CNMA model with extra interaction terms between components to allow their combination to lead to larger/smaller effects than the sum of their effects [27, 34, 35]. Allowing a clinically meaningful interaction between two (or more, e.g., three-way interaction) components, the interaction model allows components to act synergistically or antagonistically. The relative effect of an intervention comprising components *A* and *B* versus intervention C is *d*_*A*+*B vs C*_ = *d*_*A*_+*d*_*B*_+*d*_A*B_−*d*_*C*_, where *d*_*t*1*t2_ the effect of interaction between interventions *t*1 and *t*2. The assessment of the assumption with interaction can be tested using likelihood ratio tests [33]. Interaction terms should be defined a-priori [35, 56]. A currently developed method for model selection on CNMA models can be used on top of this prespecification to decide which interactions to include in the CNMA model and therefore which CNMA model fits best [38].

In the panic disorder example, several clinically relevant interaction terms can be examined. For presentation issues, we only show the case of including the interaction between the *psychoeducation* and *interoceptive exposure* component in the model. Allowing the interaction between *psychoeducation* and *interoceptive exposure* component, we can estimate the interaction effect of *phycoeducation*(*pe*)*interocaptive exposure(*ine*). Then, the relative intervention effect for interaction CNMA model for intervention *pl+ftf+pe+ps+ive+ine* versus *wl* is *d*_*pl*+*ftf*+*pe*+*ps*+*ive*+*ine vs wl*_ = *d*_*pl*_+*d*_*ftf*_+*d*_*pe*_+*d*_*ps*_+*d*_*ive*_+*d*_*ine*_+*d*_pe*ine_−*d*_*wl*_.

### Additive and interaction CNMA model in practice

Splitting interventions into different components provides new methodological challenges. There are still outstanding methodological issues with implementing additive and interaction CNMA models. Methods for testing consistency in standard NMA need to be expanded for CNMA models. Additionally, a methodological extension for the plausibility of assumptions behind CNMA models for disconnected networks is required. Additivity and interaction CNMA models need to be extended when a disconnected network at the component level is provided *without having common components*. Ranking measures and other methods from standard NMA need to be tailored for CNMA models.

The additive and interaction CNMA models have been applied in practice in a Bayesian setting (Welton *et al*. [27], Caldwell and Welton [36], Mills *et al*. [35, 56], Freeman *et al*. [32], Pompoli *et al*. [54], Madan *et al*. [30]). Pompoli *et al*. [54] also provided an assessment of the additivity assumption in the Bayesian framework. Bayesian CNMA models can be implemented in any Bayesian software (e.g. WinBUGS [57], OpenBUGS [58], rjags [59], etc.). Rücker *et al*. have recently provided additive and interaction CNMA models in a frequentist framework [33]. The additive and interaction CNMA model can now be implemented with the R package **netmeta** [60, 61] using the commands netcomb()and discomb()for connected and disconnected networks, respectively [33, 60]. If we make an additivity assumption, it is possible (but not necessary) to assume one of the interventions to be inactive, across the network, i.e. to have no intervention effect. Using the commands for CNMAs (for example, netcomb()), there is a choice to make a distinction between this inactive intervention and the reference intervention (formally used for presenting the results, for example, a comparison in a forest plot). Readers should note, however, that there is no need to specify a reference and/or inactive intervention in order to fit the model.

**Fig 3:**
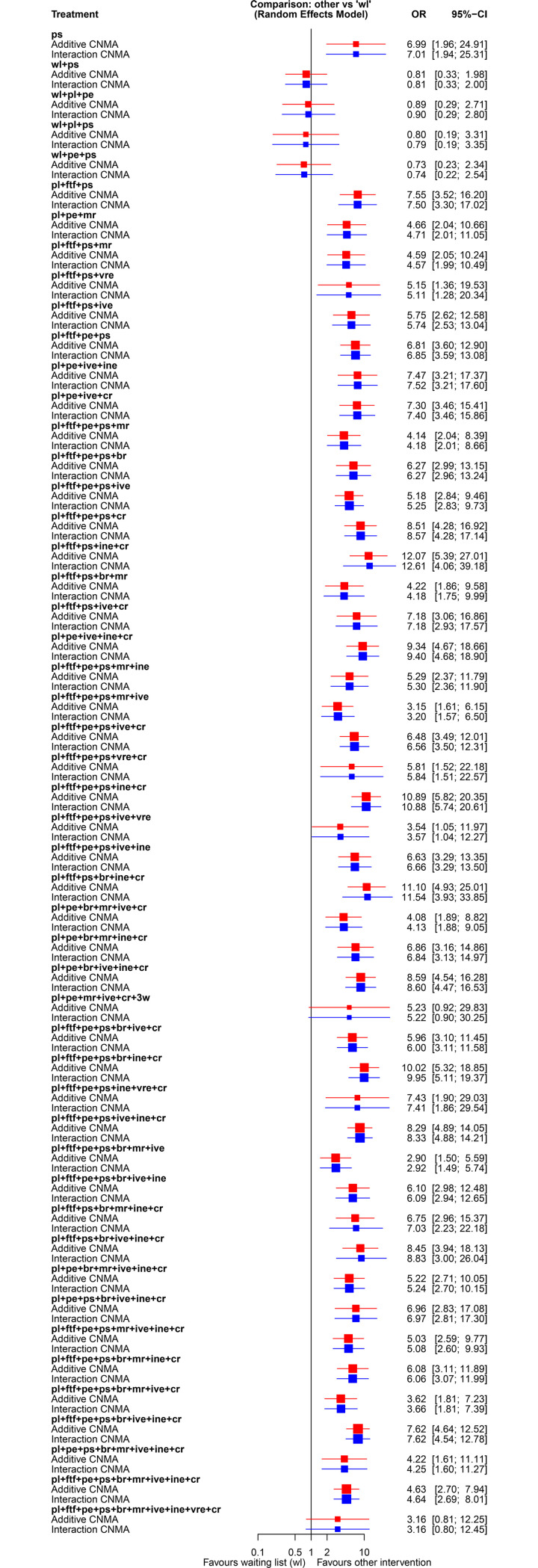
Results from fitting the additive and interaction CNMA model in the panic disorder dataset. The interaction model assumes only one interaction term between components *psychoeducation (pe)* and *interoceptive exposure (ine)*. Both analyses were conducted in the frequentist framework with the discomb() command in the **netmeta** package [33, 60]. Estimation of the combination component interventions versus the reference waiting list component (*wl*) is provided in the OR scale with their 95% confidence intervals (CI). The red color is for the additive CNMA model, blue for the interaction CNMA model.

We provide the analysis results with CNMA models for the network of interventions in panic disorder [54]. As shown in Fig 2(B), the network structure at the combination level is disconnected and it consists of three networks of interventions in panic disorder [54]. We implemented the analysis in the frequentist framework with discomb()command from **netmeta** [60, 61] package. Waiting component *wl* was used as the reference intervention for presenting results. Fig 3 presents the results in the odds ratio scale of the additive and interaction CNMA model (allowing interaction between *psychoeducation (pe)* and *interoceptive exposure (ine)*) for the network of interventions for panic disorder [54].

In general, adding the interaction between *psychoeducation (pe)* and *interoceptive exposure (ine)* did not alter the results (Fig 3). Pompoli et al. [54] examined several suspected interactions but found no strong evidence for any of them; however, this might be because the panic disorder data are sparse, and thus there is a lack of power to detect interactions. The inclusion of different combinations of components can influence the effectiveness of multicomponent therapies and explain some of the statistical heterogeneity estimated in the case of lumping. The inclusion or exclusion of each component can increase or decrease the outcome effectiveness. For instance, in the additive CNMA model, the inclusion of the *ps* component in an intervention increases the overall efficacy for remission in panic disorder by an *OR* = 6.99 [1.96; 24.91], however, there is large uncertainty around this estimate. The addition of the *pl* component combined with *ftf*, *ps*, *ine*, *cr* components (*pl+ftf +ps+ine+cr*) leads to an *OR* = 12.07 [5.39; 27.01] and therefore to the most effective combination of components for the remission in panic disorder (Fig 3).

## Conclusion

We provided an overview of meta-analytical methods used for evaluating the effects of complex interventions. Systematic reviewers should recognize the advantages and limitations of each method and define a-priori the method of analysis.

A decisive aspect of the analysis is the node-making process. There is currently a lack of guidance on reporting the process of defining nodes in NMAs. Reporting of the node-making process in published applications seems insufficient and may potentially compromise the external validity of the analyses [62]. Generally, it should be based on clinical arguments defined a priori. A panel of experts needs to identify key features of interventions and provide a relevant taxonomy that pertains to the research question.

There is an increased awareness of the methodological challenges when handling complex interventions in the meta-analytical context. We argue that this trend will continue, especially for the CNMA models, since there are recent methodological and software advances.

The additive CNMA model seems the most attractive approach, when the additivity assumption holds, as it offers the advantage to explore the comparative effectiveness of all possible combinations of components. Τhe plausibility of the additivity and interaction assumptions behind the CNMA models should be evaluated, in addition to the assumptions of the standard NMA. Methods for testing the consistency assumption, ranking measures, and methods developed for NMA may need tailoring to be employed in CNMA. Methodological extensions for the implementation of CNMA models of disconnected networks (such as additivity or consistency assumption) need to be provided. New methodological aspects of CNMA models can be further tested in real or simulated data sets.

## Supporting information

S1 Table Synthesis of studies for short term remission in panic disorder in patients with or without agoraphobia. (XLSX)

S1 File File with data and R codes. (ZIP)
